# Precision Oncology—The Quest for Evidence

**DOI:** 10.3390/jpm9030043

**Published:** 2019-09-05

**Authors:** Theodoros G. Soldatos, Sajo Kaduthanam, David B. Jackson

**Affiliations:** Molecular Health GmbH, 69115 Heidelberg, Germany

**Keywords:** clinical trial design, personalized cancer medicine, genome-based diagnostics, clinical utility, treatment decision support

## Abstract

The molecular characterization of patient tumors provides a rational and highly promising approach for guiding oncologists in treatment decision-making. Notwithstanding, genomic medicine still remains in its infancy, with innovators and early adopters continuing to carry a significant portion of the clinical and financial risk. Numerous innovative precision oncology trials have emerged globally to address the associated need for evidence of clinical utility. These studies seek to capitalize on the power of predictive biomarkers and/or treatment decision support analytics, to expeditiously and cost-effectively demonstrate the positive impact of these technologies on drug resistance/response, patient survival, and/or quality of life. Here, we discuss the molecular foundations of these approaches and highlight the diversity of innovative trial strategies that are capitalizing on this emergent knowledge. We conclude that, as increasing volumes of clinico-molecular outcomes data become available, in future, we will begin to transition away from expert systems for treatment decision support (TDS), towards the power of AI-assisted TDS—an evolution that may truly revolutionize the nature and success of cancer patient care.

## 1. Introduction

The advent of genomic technologies, such as next generation sequencing (NGS), has allowed for industry and academia to increasingly offer products and services that enable physicians to understand the therapeutic implications of patient data, based on the computational analysis of real-time clinical and molecular knowledge. While this information-driven approach is widely believed to define the future of cancer patient care, conclusive evidence demonstrating the broad clinical utility of this strategy is still lacking. The result is an environment where innovators continue to accrue significant financial burden and risk, whilst the payers and regulators await the delivery of applicable evidence. It is therefore incumbent on pharmaceutical companies, diagnostics companies and providers of decision support analytics, to expeditiously produce the evidence that is required to demonstrate the positive impact of these technologies on patient survival and/or quality of life. From a commercial perspective freedom to operate and viable business models are all dependent on the success of these endeavors—for patients, it is the perspective of improved effectiveness and decreased resistance to anti-cancer therapies that promises to positively impact the overall survival and quality of life, all being informed by data coming from their own genomes.

Here, we provide a synopsis of these efforts, with the goal of providing the reader with a broad appreciation of the molecular foundations, diagnostic processes, and clinical trial approaches that are currently defining the field. We explain the range of actionable biomarker types that can be gleaned from NGS data and highlight some of the key technologies and clinical studies that are helping to clarify the clinical utility of these innovations in evidence-based medicine.

## 2. From Drug Mode of Action to Precision Oncology

**Figure 1:**
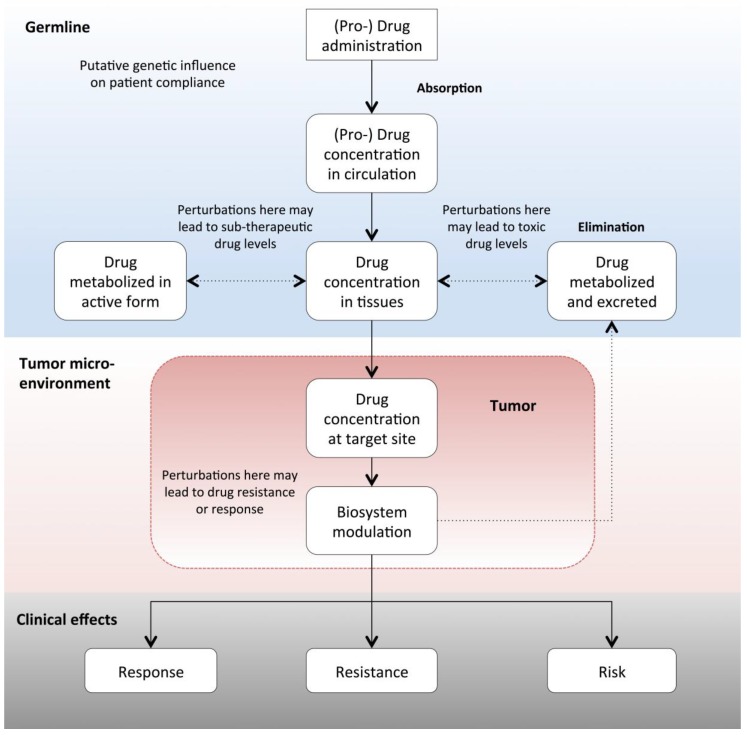
Schematic overview of the molecular path followed by orally administered anti-cancer drugs and the biological contexts in which genomic variation can affect treatment response, resistance and/or risk/safety. Factors affecting drug resistance can occur at any level, even potentially starting with patient compliance. Non-somatic mutations can affect drug pharmacokinetics leading to critical variations in available concentrations of active drug species, often associated with sub-therapeutic (i.e., drug resistance) or supra-therapeutic (i.e., drug toxicity) levels of drug concentration. Tumor specific genomic variation not only influences the pharmacodynamics of drug treatment at an intrinsic level, but variants such as tumor mutational burden can also extrinsically influence the interaction between the tumor and its microenvironment. It is primarily the sum of all these potential effects that defines clinical outcomes in response to treatment with anti-cancer therapies.

Once a drug is administered to a patient, it begins a complex journey involving diverse molecular interactions in numerous biological contexts (see Figure 1). These molecular liaisons are not only essential for the arrival/removal of an active drug to the diseased system, but they are also directly responsible for the clinical effects of drug response, resistance and risk. System perturbations, primarily in the form of genetic variation, are the primary determinants of the differential clinical outcomes to drug treatment that was seen across patient populations. Understanding the nature of these effects is key to deriving clinical utility in the form of predictive biomarker tests. Given that cancer is the most genetically heterogeneous of all diseases, a key question facing oncologists today is “how can I use this knowledge to choose the treatment(s) that provides my patient with the highest likelihood of curative outcome and minimal likelihood of drug resistance and toxicity?” The answer to this question lies in understanding the relationship between the molecular composition of the patient/tumor and the existing evidence of its clinical utility. Today, efforts to decipher such relationships provide the cornerstones of innovation in personalized medicine.

Three major classes of predictive biomarkers are currently driving the emerging practice of precision oncology. The first relies on the biological principle of oncogene addiction [1]—the observed dependency of tumor cells on cancer driver mutations (CDM’s). CDM’s typically usurp the mechanisms that control cellular signaling, which often leads to the constitutive activation of cellular growth and survival pathways. The functional dominance of such mutations ensures that CDM’s are not only prime targets for therapeutic intervention, but they also serve as excellent predictive biomarkers. In terms of aberration type, they are most commonly observed as single nucleotide variants (SNV’s), insertions/deletions (InDel’s), and fusion proteins (for a sample of biomarkers approved for clinical decision making by the US FDA, see [2,3]). Beyond the tumor specific effects on drug response, such variants can also affect toxicity to anti-cancer therapies when present as germline polymorphism.

More recently, a second class of predictive biomarkers has appeared that acts by influencing the sensitivity of a tumor to immune recognition. Perturbations in mismatch repair genes, for example, often referred to as deficient mismatch repair (dMMR), can be deleterious to genomic stability and loss of function mutations in associated genes are known to correlate with high rates of tumor mutational burden (TMB) and microsatellite instability (MSI). Tumors that have higher levels of such mutations are believed to express more neoantigens—a type of cancer-specific antigen—that may allow for a more robust immune detection, which results in more durable response to immune checkpoint inhibitors, such as Pembrolizumab. The FDA approved Pembrolizumab that is based on cumulative data from five clinical trials totaling 149 patients, for tumors with high levels of MSI [4]. This tumor agnostic approval of Pembrolizumab was the first of its kind for a predictive cancer biomarker, which is a precedent that has recently been followed by a second cancer agnostic approval of Larotrectinib in patients with solid tumors harboring *NTRK* gene fusions [5].

Perturbations in the DNA damage repair machinery also lend themselves to another distinct therapeutic strategy that is based on the concept of synthetic lethality. Synthetic lethality provides the third class of predictive biomarker and it is based on the fact that cell death is more efficiently induced by the simultaneous loss of function of two or more key players in cellular signaling pathways. The most successful example involves the clinical use of PARP inhibitors in patients with deleterious or suspected deleterious germline mutations in the *BRCA1/2* genes [6]. Current work is investigating the predictive relevance of other genes involved in homologous recombination DNA repair, such as *ATM*, *ATR*, *PALB2*, *RAD51*, *CHEK1,* and *CHEK2*, raising hopes that greater predictive resolution can be achieved, particularly for hereditary breast and ovarian cancer patients, through detailed analysis of their broader mutational profile.

### 2.1. The Case for Multi-Gene Diagnostic Testing

**Figure 2:**
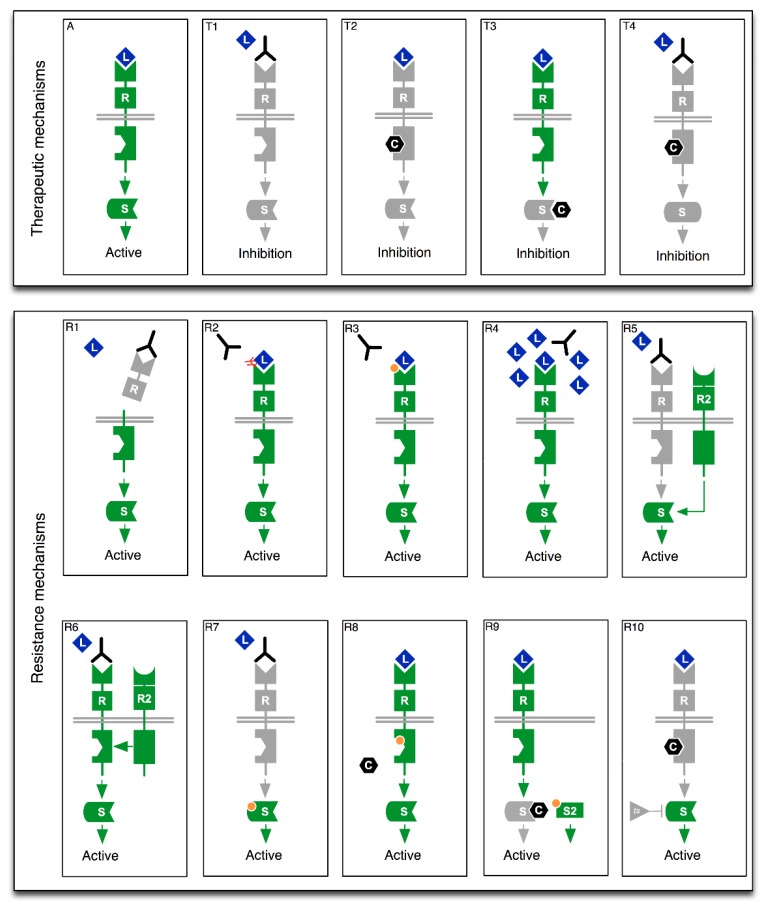
Schematic overview of select therapeutic strategies (**T1**–**T4**) and associated resistance mechanisms (**R1**–**R10**). (**A**). Binding of ligand (L) to receptor (R) induces active cellular signaling. (**T1**): Antibody based therapies can block ligand induced signaling. (**T2**): Small molecules (C) can also directly inhibit receptor kinase domain activation. (**T3**): Downstream signaling molecules can also be inhibited by small molecule chemistries. (**T4**): Combination approaches involving antibodies and small molecules can also be used. (**R1**): Ecto-domain shedding of antibody epitope [9]. (**R2**): Epitope masking [10]. (**R3**): Mutation in Antibody binding domain. (**R4**): Competitive inhibition of antibody binding via ligand overexpression [11]. (**R5**): Activation of downstream protein via alternative receptor [12]. (**R6**): Transactivation of antibody bound receptor by an alternative receptor [13,14]. (**R7**): Activating mutation in downstream protein [15,16]. (**R8**): Mutation in small molecule drug binding domain [17,18]. (**R9**): Signal activation via alternative cytoplasmic signaling protein [8,19]. (**R10**): Down-regulation of a tumor suppressor leading to activation of downstream signaling [20,21]. Green state indicates active molecule, grey state indicates inactive molecule and orange circles indicate point mutations.

Taken together, such diverse sources of predictive biomarker knowledge emphasize the importance of transcending traditional single gene testing in favor of more comprehensive diagnostic strategies. Multi-gene testing provides critical context to biomarker findings beyond the longer-term discovery implications and optimized use of valuable and often scarce tumor samples, which ensures that key information is not omitted from the treatment decision process. This is particularly important in cases where multiple independent biomarkers predict different responses to the same therapy. The literature is replete with a diversity of drug resistance mechanisms that can negate single gene diagnostic findings (for an overview of some of these mechanisms, see Figure 2). For example, Erlotinib is associated with the prolongation of progression-free survival (PFS) in non-small cell lung cancer (NSCLC) patients with certain *EGFR* mutations, but with a shortening of PFS in patients with concurrent *KRAS* mutations [7].

Given such knowledge, it would be medically shortsighted to test a lung cancer patient for mutations in only one of these genes. Another early example comes from melanomas, where *BRAF* V600E mutations are common. Nazarian et al. [8] have shown that certain activating mutations in *NRAS* (e.g., Q61K) and other upstream components, such as *PDGFR*β, can lead to resistance to drugs, like Vemurafenib and Dabrafenib.

Identifying such “predictive modifier” mutations is critical in understanding the clinical implications of functional dependencies amongst detected mutations. Not only is this important for the identification of potential resistance mechanisms, but it also opens important opportunities for the rational design of personalized combinatorial treatments. For example, the ability to probe both TMB status and identify CDM’s may allow for physicians to rationally define evidence-based combination therapies that involve both immune checkpoint inhibitors, such as Pembrolizumab, and targeted agents, such as Erlotinib. Such rationally designed combinations of immunotherapies and targeted therapies are likely to become some of the most exciting areas of progress in the utility of tumor NGS data over the coming years.

### 2.2. Cancer Gene Panels and Analytical Software

Today, the most common clinical approach for multi-gene testing involves targeted gene panels. Targeted panels range from small, typically tumor-specific gene panels that start from circa ten genes, up to larger more tumor agnostic panels covering hundreds of genes (see Table 1). While whole exome sequencing (WES) provides a more comprehensive view on the tumor genome it suffers important practical limitations when compared to targeted panels. Targeted panels permit a higher sequencing depth and are still more cost-effective as well as offer faster turn-around times than WES. These simple features contribute significantly to their basic clinical applicability, a fact that was recently validated by the FDA-approval of two independent NGS tests for solid tumors: Foundation Medicine’s F1CDx (324 genes), which has been approved as a companion diagnostic, and Memorial Sloan Kettering Cancer Center’s (MSKCC) MSK-IMPACT profiling test assay (468 genes). One of the key advantages of larger gene panels is the tumor agnostic nature of their clinical utility, which typically allows for any solid tumor type to be profiled in a single diagnostic assay.

**Figure 3:**
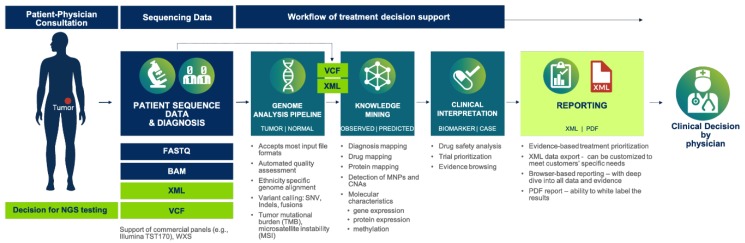
Showing the end-to-end process of genome-guided treatment decision support. After DNA sequencing of the tumor biopsy, sequence data is input to a computational system that identifies and interprets the clinical implications of genomic variants. A diagnostic report is then automatically generated and provided to the treating oncologist for review.

In terms of clinical practice, the process of NGS-driven treatment decision support (TDS) begins with a patient-physician consultation. Once NGS testing is warranted, a tumor sample and/or a matched normal sample, together with a test requisition form, is sent to a diagnostic lab for sequencing. The resulting sequence data is uploaded into a genome informatics/TDS software, such as MH Guide (www.molecularhealth.com), which then identifies and clinically interprets genomic variants, such as SNV’s, gene fusions, InDel’s, copy number alterations (CNA’s), TMB, or MSI. These systems function by comparing the identified variants against curated knowledge bases containing comprehensive information regarding approved and emergent predictive biomarkers. The findings are summarized in a concise report, allowing for physicians to quickly review current knowledge around the treatment implications of a variant, and the evidence of its clinical utility in the current patient’s case. Such reports automatically present the most clinically relevant biomarker knowledge first, prioritized in terms of the degree of available evidence (Figure 3).

Today, the process of automated clinical interpretation is made possible through expert curated databases of predictive biomarker information. The capture and curation of this knowledge by biomedical experts is critical to the entire process and it serves a number of important roles. It ensures that all information regarding the effects of a biomarker, including validation and reliability levels, is captured in an accurate manner. Such quality and evidence measures are directly reported to clinicians in the diagnostic report, ensuring that they are explicitly clear about how relevant a biomarker finding might be for each patient. The aforementioned MH Guide system for example, employs three quality levels of clinical validity:

**QUALITY LEVEL 1: Clinically endorsed** biomarkers (i.e., recommended by key opinion leaders such as ASCO, EORTC, NCCN, ESMO, FDA, EMEA). This is considered as the information of highest relevance.

**QUALITY LEVEL 2: Clinically observed** biomarkers (i.e., observations coming from real patient data but not yet endorsed by a key opinion leader—including prospective, retrospective, registries, meta-analysis or n-of-one studies). This is considered as information of high relevance.

**QUALITY LEVEL 3: Pre-clinically observed** biomarkers observed in vitro or in vivo animal model systems or predicted in silico. This is considered as information of lower/unclear relevance.

Other essential information may also be captured during the curation/reporting process, including:

(a) Detailed variant descriptions—e.g., the type of genomic aberration (such as SNP, Insertion, or Deletion, etc.)
(b) The relevant drug or treatment
(c) The effect of variant on treatment responsiveness—i.e., response, resistance or toxicity
(d) The quantity of effect—e.g., strong, medium, weak
(e) The observation context (i.e., the disease, disease stage, or model system)
(f) A link to the source information and a grading of its reliability

By covering a broad spectrum of evidence levels, from emergent to clinically approved, the TDS systems facilitate the increasingly daunting task of deciphering whether identified biomarkers and associated therapies are likely to benefit a given patient, especially where more than one biomarker is detected. Such “expert systems” make this information available to physicians, which enable them to ascertain whether a variant was previously observed in similar patient cases, together with the observed clinical effects of drugs used in these contexts. While information about emergent biomarkers is certainly of lesser clinical utility than clinically endorsed biomarkers, they can provide an instructive data-point for clinicians treating late-stage cancer patients where no obvious treatment options exist. In such contexts, the information is often used to prioritize the clinical trial options for the patient. Given that predictive biomarker information tends to be disease specific, individual variants can have differential levels of both predictive power and evidence as one moves from one indication to another. This highlights the fact that the more molecular outcomes data we generate around biomarkers, the greater the challenge for physicians to clinically interpret a patient’s mutational profile becomes. The flipside is that as more clinico-molecular outcomes data is generated globally, the more we will be able to capitalize on the power of AI. This is an evolution that will decrease our current use of expert systems, which allows us to glean new knowledge and treatment pathways from real world clinical and molecular outcomes data.

However, today there are several challenges that still impede the effective application of AI in TDS. On the one level, it is important that we remain objective about the current applicability of the technology in the treatment setting. While machine-/deep-learning has shown great promise in areas, such as drug discovery and the automated analysis of diagnostic images [22], it is still too early for broad reliable application in treatment decision making across multiple cancer types. This point is best illustrated by the challenges that are faced by the IBM Watson technology in this specific setting, most likely due to the lack of available clinico-molecular outcomes data upon which the system could “learn”. Access to such data, preferably for millions of patients, still remains a prerequisite for the reliable application of AI in genome guided treatment decision-making and we still face considerable challenges, especially with respect to data access, standardization, and privacy. Such technology, regulatory, and ethical issues, together hinder the broad community-wide availability of such data, and until these problems are effectively addressed, expert TDS systems will continue to play a dominant role in providing—and proving—the clinical utility of the genome-driven approaches to TDS.

## 3. Precision Oncology Trials—The Quest for Evidence

Clinical utility is broadly defined as a measurable improvement in patient outcomes based on patient management changes that are directed by the result of the test [23]. It represents the final and most important of three key parameters from the ACCE model, which takes its name from the criteria for evaluating a genetic test—analytic validity, clinical validity, clinical utility, and associated ethical, legal, and social implications. The ACCE framework has guided or has been adopted by various entities in the US and worldwide for evaluating genetic tests in a format that provides policy makers with current and reliable information for decision-making. Despite the enormous promise of genome-driven TDS, we still await conclusive evidence of its broad clinical utility and specific impact on patient outcomes. In the absence of such evidence, payers have maintained a modus of “show-me-the-evidence”, and this stance is likely to remain until such evidence is forthcoming. In determining whether to cover new technologies and diagnostic tests, payers are likely to consider the following questions:

- What is the strength of the clinical evidence that the technology is safe and effective?
- What group of patients, if any, would benefit most from using a given technology for preventing, diagnosing, or treating a particular condition?
- Under what circumstances and conditions, if any, would the technology be most appropriately used?
- How does the new technology compare to other available treatments for the same condition?

In addition to the payer perspective, regulatory bodies are also looking at closer regulation of the entire cancer diagnostics space, which further heightens the need for evidence of clinical utility. In the following section, we look at some of the key endeavors in the quest to generate this much needed evidence base.

### 3.1. Clinical Trial Strategies

**Figure 4:**
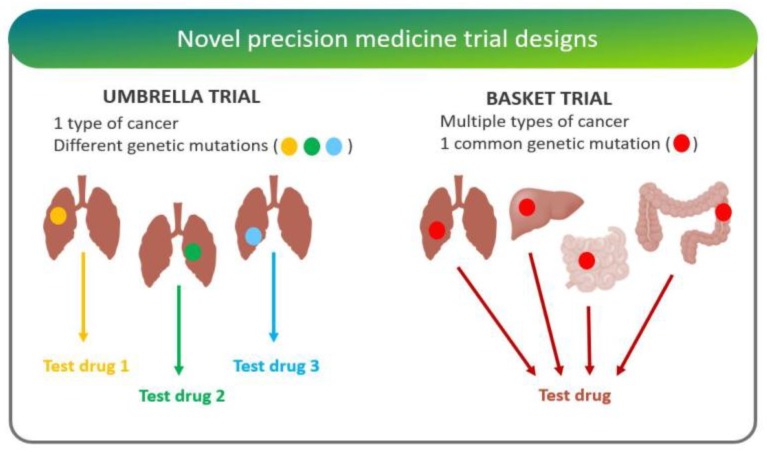
Depicting the core difference between “umbrella” and “basket” trial designs—figure adapted from [27]. Umbrella trials assign patients with the same type of cancer to different arms based on their mutations. Basket trials, on the other hand, test whether a drug is effective in patients with specific genetic alterations regardless of their disease of origin.

A diversity of trial strategies has emerged over the past number of years, aimed at quantifying the degree of clinical utility that was provided by multiplexed gene testing. While Phase III randomized trials are the gold standard for approval of novel interventions, these studies are complicated and expensive. For this reason, studies around the clinical utility of gene testing tend to involve innovative phase II trials, with designs that include randomization, adaptive design, basket, umbrella, and n-of-one studies (Figure 4).

- “Adaptive trials” evolve dynamically based on emergent trial data. This leads to hypotheses optimization and testing where randomization ratios can be modified, treatment arms with inferior outcomes eliminated, and increased biomarker-based assignment results in a higher proportion of patients to be randomly assigned to the more effective treatment arms [24].
- “Basket trials” test whether a drug is effective in patients with specific genetic alterations regardless of their disease of origin [25]. For instance, the National Cancer Institute Molecular Analysis for Therapy Choice (NCI-MATCH) is a Phase II clinical trial in which patients who share a common genetic mutation for a given cancer are sorted into “baskets”, or treatment arms, regardless of cancer type.
- “Umbrella trials” assign patients to one of potentially many treatment arms, based on a specific cancer type and genetic markers. For example, the Lung Master Protocol (Lung-MAP) study aims to rapidly identify drug therapies for particular cancer types, making it a useful design for cancers with wide genetic heterogeneity [24].
- In “n-of-one trials”, the patient serves as both control and experimental “arm”. This design is especially useful for low-frequency molecular aberrations/conditions, where randomized studies are difficult [26].

PFS is the most common clinical metric applied in such studies that can in turn be divided into four basic trial types, each of which is discussed in greater detail in the subsequent sections:

- Randomized/Histology-agnostic
- Randomized/Histology-specific
- Non-randomized/Histology-agnostic
- Non-randomized/Histology-specific

### 3.2. Randomized Histology-Agnostic Studies

Two major trials exploring the clinical utility of multiplexed gene testing in a randomized, histology agnostic manner are the IMPACT II and MPACT studies (see Table 2). IMPACT II is different, in that it is a single blind study with parallel assignment intervention model, as opposed to the open label MPACT trial that uses a crossover assignment intervention model.

#### 3.2.1. IMPACT II (MD Anderson Cancer Center/Foundation Medicine)

The official title of this study is “Randomized Study Evaluating Molecular Profiling and Targeted Agents in Metastatic Cancer: Initiative for Molecular Profiling and Advanced Cancer Therapy (IMPACT 2)”. Its primary objective is comparison of PFS between two randomized arms, or more explicitly, to determine whether the patients treated with a targeted therapy selected on the basis of a genomic tumor aberration have increased PFS as compared to those who are not.

#### 3.2.2. M-PACT (NCI)

The official title of this study is “Molecular Profiling-Based Assignment of Cancer Therapy for Patients with Advanced Solid Tumors”. Its primary objective is to assess the utility of genome sequencing to determine therapy and improve patient outcomes in early-phase trials, independent of tumor type. The endpoint of the M-PACT (Molecular Profıling-based Assignment of Cancer Therapeutics) pilot study is to assess whether the objective response rate and/or the four-month PFS is improved following treatment with agents selected based on the presence of specifıc mutations in tumors of patients.

### 3.3. Randomized Histology-Specific Studies

These umbrella studies take patients with a specific cancer type and assign them to randomized sub-trials/groups that are based on their molecular profile (see Table 3).

#### 3.3.1. BATTLE2 (MD Anderson Cancer Center)

The BATTLE-2 program is a targeted therapy study that recruited previously treated patients with advanced stage, treatment refractory NSCLC. The primary objectives are to determine the eight-week disease control rate, and to learn whether drug or drug combinations that are based on a patient’s biomarker profile can improve outcomes, in four treatment arms. The study is conducted in two stages. In stage I, the patients were adaptively randomly assigned based on their *KRAS* status [28]. In stage II, an additional equal number of patients are to be adaptively assigned based on biomarkers selected from stage I [29].

#### 3.3.2. ALCHEMIST (NCI)

The “Adjuvant Lung Cancer Enrichment Marker Identification and Sequencing Trial” (ALCHEMIST) studies the impact of genetic testing in patients with stage IB-IIIA NSCLC. Patients with a mutation targeted by one or more of the investigational drugs used in this study (Erlotinib, Crizotinib, Nivolumab) or those without mutations are assigned to one of three randomized treatment sub-protocols.

#### 3.3.3. LungMAP (SWOG1400)

The Lung Cancer Master Protocol (official name: “A Biomarker-Driven Master Protocol for Previously Treated Squamous Cell Lung Cancer”), or Lung-MAP (SWOG S1400), is a multi-drug, multi-arm, biomarker-driven phase II/III clinical trial in patients with squamous cell lung cancer (recurrent, stage IV). The trial uses genomic profiling to match patients to investigational treatments that might target the genomic alterations, or mutations, found to be driving the growth of their cancer. Patients with genetic alterations are randomly assigned to receive investigational, targeted therapy versus standard therapy. Instead of having to undergo multiple diagnostic tests to determine eligibility for many different studies, enrollees are tested just once according to the “master protocol” and, based on the results of this screening, patients are assigned to one of the different trial arms (sub-studies) best suiting their genomic profile, each testing different investigational drug regimens.

#### 3.3.4. SAFIR-02 (Lung)

This is a randomized phase II trial in metastatic NSCLC patients performed in a multicenter setting. It is an open-label trial that uses high throughput genome analysis as a therapeutic decision tool, comparing a medical treatment that was administered according to the identified molecular anomaly of the tumor with a medical treatment administered without considering the tumor genome analysis. It consists of two sub-studies, one for targeted therapies and one for immunotherapy, each being compared to the respective standard maintenance therapy.

#### 3.3.5. SAFIR-02 (Breast)

This is a randomized phase II trial in a multicenter setting in patients with metastatic breast cancer and follows the same trial design principle as SAFIR02 (lung). Both SAFIR02 trials (lung/breast) aim to measure the efficacy of genome analysis as a therapeutic decision tool for patients. 

### 3.4. Non-Randomized Histology-Agnostic Studies

Here, we highlight four trials exploring the clinical utility of multiplexed gene testing in an open label, non-randomized histology-agnostic manner: the MATCH, WINtherapeutics, MOSCATO, and TAPUR trials (Table 4).

#### 3.4.1. MATCH (NCI)

The “Molecular Analysis for Therapy Choice (MATCH)” study includes an umbrella protocol for multiple small single-arm treatment sub-protocols that enrolls adults with advanced solid tumors, refractory cancers, lymphomas, and multiple myelomas no longer responding to standard therapy. Patients undergo biopsy, along with molecular characterization of the biopsy material for specific, pre-defined mutations, amplifications, or translocations of interest via tumor sequencing and immunohistochemistry. Consenting patients also undergo a collection of blood samples for research purposes. Additionally, WES at baseline and at the time of progression are performed for research purposes with the expectation of providing more comprehensive genomic data than would typically be available if genotyping were limited to one or more mutations known to be associated with a particular cancer type.

#### 3.4.2. WINtherapeutics

This is an open non-randomized study that uses biology-driven selection of therapies. The aim is to provide a rational personalized therapeutic choice to all patients enrolled in the study, harboring oncogenic events (such as mutations, translocations, or amplifications) or not. Patients enroll in the study across a number of participating cancer centers. WINtherapeutics (or WINTHER) explores matched tumor and normal tissue biopsies and uses a novel method for predicting efficacy of drugs: given that patients entering in each trial arm were treated upon disease progression following the standard therapy, the trial endpoint was therefore set to be a comparison of the PFS within the trial (PFS2) with the PFS of the last therapeutic line given prior to trial entrance and treatment (PFS1), with the aim of achieving a PFS2/PFS1 ratio that is greater than 1.5 in 40% of patients.

#### 3.4.3. MOSCATO

The MOSCATO (Molecular Screening for Cancer Treatment Optimization) study is a prospective clinical trial that evaluates the clinical benefit of high-throughput genomic analyses in improving the outcomes in patients with advanced, hard-to-treat, treatment resistant metastatic cancers. The primary objective is to evaluate clinical benefit as measured by PFS on matched therapy (PFS2 using a targeted treatment selected by molecular profiling) as compared to the PFS on most recent prior therapy (PFS1). Thus, both MOSCATO and WINTHER employ similar metrics to evaluate the benefit of molecular screening analysis as decision tool for targeted molecular treatment in patients with difficult, non-curable metastatic cancers.

#### 3.4.4. TAPUR

The “Targeted Agent and Profiling Utilization Registry” (TAPUR) Study is a non-randomized open label clinical trial that aims to describe the safety and efficacy of commercially available, targeted anticancer drugs that are prescribed for treatment of patients with advanced cancer, whose tumor harbors a genomic variant that is known to belong to a drug target or to predict sensitivity to a drug. TAPUR is currently recruiting—its main intention is to learn directly from real world practice about the prescribing to patients of FDA approved targeted therapies as well as catalogue the choice of molecular profiling test by clinical oncologists.

### 3.5. Non-Randomized Histology-Specific Studies

Last, we describe two non-randomized histology specific (breast cancer) studies that preceded the randomized SAFIR-02 histology specific (lung and breast) trials, namely the Pre-SAFIR and the SAFIR-01.

#### 3.5.1. Pre-SAFIR

Samples from metastatic breast cancer patients were prospectively or retrospectively collected from frozen or paraffın-embedded tissue and were then analyzed. This trial showed that genetic testing (such as comparative genomic hybridization array) is feasible in the context of daily practice and, in combination with *PIK3CA*/*AKT1* mutation assessments, identified a substantial number of actionable mutations that allow for matching with specific targeted agents.

#### 3.5.2. SAFIR-01

In this study, breast cancer patients with metastasis accessible to biopsy were studied and offered targeted therapy based on identified genomic actionable mutations. This study suggested that personalized medicine for metastatic breast cancer is feasible and confirmed the high prevalence of rare targetable genomic alterations. This study was important, in that it also highlighted the need to secure better access to relevant drugs in personalized medicine programs, something that is a major consideration with all such trials.

### 3.6. Perspectives

To date, a number of prototypical precision oncology trials have been completed, leading to a promising, although still immature, evidence base. The first was reported in 2010 by von Hoff and colleagues [30], demonstrating a longer PFS for 27% of patients receiving biomarker directed therapy when compared to clinician choice. This multi-center trial, performed across nine different cancer centers in the US, used a novel study design in which the patients served as their own controls—a kind of “multi n-of-one” study. Here, the time-to-progression (TTP) ratio was determined by actual comparison of TTP on biomarker-guided therapy versus the TTP on the patient’s prior therapy. For the participants who had a PFS ≥ 1.3, the overall survival was 9.7 months as compared to five months on physician directed therapy. Interestingly, multi-biomarker testing of tumors yielded actionable targets in 98% by this assay, which indicated for the first time that such approaches were feasible in the clinic. Moreover, the study design has inspired similar approaches in the aforementioned MOSCATO and WINTHER trials.

Instructive results from the IMPACT I trial, a predecessor of the currently ongoing IMPACT II trial, have also been reported. Here, Tsimberidou L. et al. [31] used a multi-gene panel to analyze patients with metastatic or inoperable cancer. Clinical outcomes of patients with gene aberrations that were treated with matched therapy were then compared with those patients with gene aberrations who were not treated with matched therapy (due to issues, such as: eligibility, study availability, insurance coverage, and/or logistical problems). Bearing in mind that the study was not randomized and patients had diverse tumor types and a median of five prior therapies, their results also suggested that identifying specific molecular abnormalities and choosing therapy that is based on these abnormalities is relevant, at least in the treatment of late stage cancer patients who are eligible for phase I study. More recent comprehensive results from the study were reported and confirm that use of tumor molecular profiling matched with targeted therapy associates with encouraging rates of response, PFS and overall survival as compared to non-matched therapy [32]. To further evaluate molecular profiling and targeted agents in patients with metastatic cancer, IMPACT II was recently initiated, this time conducted as a randomized trial aiming to compare treatment with and without genomic selection [32,33].

Despite the positive indications from these initial studies, controversy has also emerged from the SHIVA trial [34]. This was an open-label, randomized, controlled phase II trial, which was performed at eight academic centers. The results from this study failed to show a positive difference in PFS between its two groups, a fact likely attributable to several limitations that are associated with the trial design. For example, inaccuracy in some of the matches between the variants and targeted treatments highlighted how important comprehensive TDS systems are in aiding evidence-based treatment prioritization [35]. While considering such critical shortcomings, it is unfortunately not possible to use results from the SHIVA study to derive definitive conclusions about the clinical utility of genomic profiling in cancer patient care. Notwithstanding, the study has shown the feasibility of performing a multi-center phase II trial employing genomic profiling technologies, a fact that, in itself, has been helpful in evolving clinical trial strategy in the field.

Each of these preliminary results suggest that, in early clinical trials, matching patients with targeted drugs based on their molecular profile results in (a) longer response times when compared to their prior therapy and (b) higher rates of response, survival, and time to treatment failure as compared to those that were seen in patients treated without molecular matching. They also emphasize the limitations of the “one-biomarker—one-drug” approach, suggesting that multi-biomarker testing of tumors at the time of diagnosis, and subsequently at all points of tumor recurrence, whether local or distant, will in the future bring enhanced clinical benefits, in the form of improved survival and quality of life. Together, these early results suggest that the clinical success of these approaches will ultimately also be determined by factors, such as the:

1. Number of genes characterized
2. Extent of gene characterization (sub-exome, exome, or whole genome)
3. Ability to computationally interpret the clinical implications of patients’ clinical and molecular data
4. Clinical experience of the treating physician(s)
5. Availability of prioritized therapies

While there is a tendency in the field to equate the diagnostic power of a test with the number of genes included, this perspective ignores the critical importance of the TDS software. In fact, it is quite reasonable to assume that a 50-gene test might outperform a 500-gene test, if the former is connected to a more powerful TDS platform. After all, it is the ability to access and interpret clinico-molecular evidence, combined with our ability to identify genetic variants, which should define the clinical utility of the precision oncology process. Moreover, beyond identifying the actionable mechanisms of drug resistance and response, TDS platforms can also provide important insights into toxicity considerations, whether they are polymorphism-based or pharmacologically driven (i.e., drug interactions). Indeed, during the so-called PePaCaKa-01 trial (NCT02767700), a 600+ gene panel was used in combination with the MH Guide platform to detect toxicity biomarkers in 27 out of 31 patients: first/ongoing data analysis showed that 13 of the 27 received chemotherapy for which they had a relevant toxicity marker, while seven of the 13 had a biomarker that could have predicted the toxicity in a prospective setting [36]. These results demonstrate the exciting potential of using NGS based testing in the prediction of drug side effects, which is critical from a cost and quality of life perspective, and a follow-up to PePaCaKa-01 study is planned to further explore such utility. Meta-analyses have shown that treatment-related mortality rate and toxicity was lower when a personalized approach was used, despite the fact that quality of life has not been regularly assessed in some molecular profiling studies [37,38].

## 4. Discussion

Precision oncology is, in essence, a data-driven approach to optimizing cancer patient care. It relies on the search for actionable features of cancer disease mechanisms, such as (1) oncogene addiction—tractable using targeted therapies and (2) deficiencies in DNA repair/recombination—tractable using immunotherapies, such as immune checkpoint inhibitors or the principles of synthetic lethality. Molecular technologies are thus combined with established clinical processes to generate patient-specific clinico-molecular profiles. These profiles are then compared against existing knowledge by using TDS software solutions, which automatically identify and compare tumor variants against curated databases of peer-reviewed predictive biomarker knowledge. The goal of these systems is to search for treatment strategies, previously observed in similar patients that provide the highest likelihood of treatment response and lowest likelihood of drug resistance and/or toxicity. These expert systems then prioritize the potential treatment options based on the degree of clinical evidence in a patient/disease specific manner and summarize the results in the form of a diagnostic report. These reports are geared at helping doctors address the following key questions:

- Which drugs stand the highest likelihood of working in my patient?
- Which drugs stand the highest likelihood of not working in my patient?
- Which drug combinations might be particularly efficacious?
- Which drugs and drug combinations should be contraindicated?
- What dose should a particular drug/combination be given at?

Today, a diversity of innovative trial strategies are being pursued in the search of more precise definitions of clinical utility strategy across cancer indications and disease stages. While the US has certainly been the trendsetter, precision oncology trials are now globally prevalent. Funding for precision oncology has been advanced in Europe, for example, with the advent of the EU’s Seventh Framework Program the Innovative Medicines Act and Horizon 2020 [39,40]. In addition, six major European cancer centers have formed the Cancer Core Europe initiative that aims to develop novel drugs and companion diagnostics by conducting and sharing data from next generation clinical trials [41].

While numerous challenges remain in the successful implementation of precision oncology, perhaps the most formidable is the nature of cancer itself, especially late stage disease. Here, the realities of tumor heterogeneity mean that we must bring the power of modern technologies to bear on the biological principles of tumor evolution—a hugely formidable foe. Data will be key to addressing this challenge with deep molecular/sequence data and the associated clinical outcomes required for millions of patients. Combining such datasets with the power of machine learning should, in future, provide the innovative leap that is required to truly tackle metastatic disease in late stage cancer patients. Here, we can also anticipate the emergence of specifically timed regimens of combination approaches tackling the issue of residual disease and the sub-clones responsible for disease recurrence. It is at this stage that the role of the treating physician will change forever, with the diagnosis and treatment decision routinely supported by computers, resulting in a new Era of cancer patient care. However, until this time, the field must focus on not only generating clinico-molecular outcomes data for millions of patients, but also on generating the evidence base in clinical trial settings, which are required to validate this optimistic view of the future.

## 5. Conclusions

While the application of NGS testing continues to emerge as a rational and highly promising approach for guiding treatment decisions in oncology, specific outcome metrics are still required to fully understand its broader clinical utility. To this end, numerous innovative precision oncology trial designs have emerged globally that address biological challenges, such as molecular diversity, and practical realities, such as drug availability. The enormity of the challenge at hand demands community wide collaboration that will be increasingly driven by data science and machine learning approaches in the future.

Today, the process of precision oncology relies on NGS sequencing and the use of expert TDS systems for data analysis, and reporting. However, we expect that, as more clinico-molecular outcomes data emerges through the marriage of Electronic Health Record data with molecular test results, our ability to learn from real world outcomes by using AI-assisted treatment prioritization will provide the next level of insight and innovation in the field. Aligned with this perspective, we can also anticipate that complete tumor, germline and cell-free DNA/RNA profiling and proteomics will combine with an improved understanding of the immune system and the influence of our microbiomes.

The goal in the future will be to apply all of these data assets to address the highly dynamic nature of tumor biology, in search of combinatorial approaches to drug resistance, tumor heterogeneity, residual disease, and treatment toxicity. To this end, we anticipate the further evolution of clinical trial activity in this space, where the role of computational systems increasingly comes to the fore. This could eventually lead us to an Era of “clinical singularity”, where data and technology redefine the nature and success of cancer patient care forever. However, there does remain much to be achieved until we can confidently transition from the expert TDS systems of today, to the true application of AI in treatment decision support.

## Figures and Tables

**Table 1:** Sample selection of key next generation sequencing (NGS) panels.

NGS Panel Name	Number of Genes	Histology	Provider
EngineusPANEL 600+	600+	Agnostic	Molecular Health GmbH
MSK-IMPACT ^+^	468	Agnostic	Memorial Sloan Kettering Cancer Center
FoundationOne CDx ^o^	324	Agnostic ^C1^	Foundation Medicine, Inc.
Oncomine Dx Target Test ^o^	23	Specific ^C2^	ThermoFisher
FoundationFocus CDxBRCA Assay ^o^	2	Specific ^C3^	Foundation Medicine, Inc.
Praxis Extended RAS Panel ^o^	2	Specific ^C4^	Illumina

^o^ Companion diagnostic (CDx); ^+^ FDA approved; ^C1^ Cancers: Non-small cell lung, Melanoma, Breast, Colorectal, Ovarian; ^C2^ Cancers: Non-small cell lung; ^C3^ Cancers: Ovarian; ^C4^ Cancers: Colorectal.

**Table 2:** Summary of randomized histology-agnostic studies examined in this article. Data extracted from clinicaltrials.gov (October, 2018).

Name	NCT Number	Enrollment	Start	End	Condition (Disease)	Sponsor
IMPACT II	NCT02152254	391 ^A,^*	2014 ^A,+^	2020 ^E,+^	Metastatic Cancer	MDACC ^C^
MPACT	NCT01827384	700 ^E,^*	2013 ^A,+^	2019 ^E,+^	Advanced Malignant Solid Neoplasm	NCI
SHIVA	NCT01771458	742 ^A,^*	2012 ^A,+^	2016 ^E,+^	Recurrent/Metastatic Solid Tumor	Institute Curie

^A^ Actual; ^E^ Estimated (expected); * Number of participants; ^+^ Year (of respective date); ^C^ Collaborator: Foundation Medicine; MDACC: M.D. Anderson Cancer Center; NCI: National Cancer Institute.

**Table 3:** Summary of randomized histology-specific studies examined in this article. Data extracted from clinicaltrials.gov (October, 2018).

Name	NCT Number	Enrollment	Start	End	Condition (Disease)	Phase	Sponsor
BATTLE2 ^M1^	NCT01248247	334 ^A,^*	2011 ^A,+^	2019 ^E,+^	NSCLC	2	MDACC ^C1^
ALCHEMIST ^M2^	NCT02194738	8300 ^E,^*	2014 ^A,+^	2021 ^E,+^	NSCLC	3	NCI
LungMAP ^M2^	NCT02154490	10000 ^E,^*	2014 ^A,+^	2022 ^E,+^	Squamous Cell Lung Cancer	2|3	SOG ^C2^
SAFIR2 Lung ^M2^	NCT02117167	650 ^E,^*	2014 ^A,+^	2022 ^E,+^	NSCLC	2	UNICANCER ^C3^
SAFIR2 Breast ^M2^	NCT02299999	1460 ^E,^*	2014 ^A,+^	2022 ^E,+^	Breast Cancer	2	UNICANCER ^C4^

NSCLC: Non-Small Cell Lung Cancer; MDACC: M.D. Anderson Cancer Center; NCI: National Cancer Institute; SOG: Southwest Oncology Group. ^A^ Actual; ^E^ Estimated (expected); * Number of participants; ^+^ Year (of respective date). Intervention model: ^M1^ Single Group Assignment. ^M2^ Parallel Assignment. Collaborators: ^C1^ Merck Sharp & Dohme Corp., NCI, GlaxoSmithKline, Novartis. ^C2^ NCI. ^C3^ IFCT, Fondation ARC, AstraZeneca. ^C4^ Fondation ARC, AstraZeneca.

**Table 4:** Summary of non-randomized histology-agnostic studies examined in this article. Data extracted from clinicaltrials.gov (October, 2018).

Name	NCT Number	Enrollment	Start	End	Condition (Disease)
MATCH ^M1^	NCT02465060 ^S1^	6452 ^E,^*	2015 ^A,+^	2022 ^E,+^	Advanced Refractory Solid Tumors, Lymphomas, Multiple Myeloma
WINTHER ^M1^	NCT01856296 ^S2,C1^	200 ^E,^*	2013 ^A,+^	2018 ^E,+^	Metastatic Cancer, Advanced Malignancies
MOSCATO ^M2^	NCT01566019 ^S2^	1050 ^E,^*	2011 ^A,+^	2019 ^E,+^	Metastatic Solid Tumors (Any Localization)
TAPUR ^M2^	NCT02693535 ^S3,C2^	2980 ^E,^*	2016 ^A,+^	2021 ^E,+^	Advanced Solid Tumors, Lymphomas (Non-Hodgkin), Multiple Myeloma

^A^ Actual; ^E^ Estimated (expected); * Number of participants; ^+^ Year (of respective date). ^M1^ Intervention model: Parallel Assignment. ^M2^ Intervention model: Single Group Assignment. ^S1^ Sponsor: National Cancer Institute (NCI). ^S2^ Sponsor: Gustave Roussy. ^S3^ Sponsor: American Society of Clinical Oncology (ASCO). ^C1^ Collaborator: NGS performed by Foundation Medicine. ^C2^ Collaborators: AstraZeneca, Bayer, Bristol-Myers Squibb, Eli Lilly and Company, Genentech Inc., Merck Sharp & Dohme Corp., and Pfizer.
